# Pregnancy complicated with adrenal adenoma causing ACTH-independent Cushing’s syndrome, accompanied by obstetric antiphospholipid syndrome and severe pre-eclampsia: case report and literature review

**DOI:** 10.3389/fendo.2023.1147316

**Published:** 2023-05-19

**Authors:** Shenghan Xu, Miao Liu, Jiamu Xu, Bangwei Che, Wenjun Zhang, Wei Li, Tao Huang, Ying Yu, Cheng Zha, Zheng Peng, Kunyuan Huang, Kaifa Tang

**Affiliations:** ^1^ Department of Urology and Andrology, the First Affiliated Hospital of Guizhou University of Traditional Chinese Medicine, Guiyang, China; ^2^ The Clinical Medical College of Guizhou Medical University, Guiyang, China; ^3^ The Affiliated Hospital of Guizhou Medical University, Guiyang, China

**Keywords:** adrenocortical adenoma, antiphospholipid syndrome, obstetric antiphospholipid syndrome, pregnancy, cushing’s syndrome

## Abstract

This case report shares the management experience of a patient with pregnancy combined with adrenal adenoma causing ACTH-independent Cushing’s syndrome (CS), accompanied by obstetric antiphospholipid syndrome (OAPS) and severe pre-eclampsia. The case was a 26-year-old that presented with typical clinical symptoms and signs of CS. The patient had a history of 4 spontaneous abortions in the last 4 years. The 24-hour urinary free cortisol was significantly increased, an abnormal cortisol circadian rhythm was demonstrated by a high late-night salivary cortisol, blood ACTH was suppressed (< 1ng/dL), anticardiolipin antibody was positive, and imaging examination showed an adrenal tumor. The patient underwent laparoscopic adrenal tumor resection under general anesthesia at 23 weeks of gestation. The tumor was pathologically confirmed to be an adrenocortical adenoma. The patient underwent a cesarean section at 39 weeks of gestation to give birth to a healthy baby girl with an Apgar score of 10. Pregnancy complicated by CS is clinically rare, easily masked by normal physiological changes of pregnancy, and is difficult to diagnose. The determination of 24-hour urinary free cortisol, the circadian rhythm of serum cortisol, ultrasound, and MRI can be helpful in the diagnosis of CS during pregnancy. Surgery is the first choice for the treatment of CS during pregnancy. As a subtype of antiphospholipid syndrome, patients with OAPS are prone to thrombotic events and recurrent miscarriages if not treated accordingly. To our knowledge no cases of CS with OAPS and severe pre-eclampsia have been reported. We summarize the experience of the treatment of this patient and review the literature to improve clinicians’ awareness of this disease.

## 1 Introduction

Cushing’s syndrome (CS) is a rare condition due to chronic exposure to high circulating cortisol levels (1). The main clinical manifestations of CS are the full moon face, buffalo hump, centripetal obesity, purple striae, hypertension, proximal myopathy, and osteoporosis (2). Cushing’s syndrome is most common in women between the ages of 20 and 40 (2). Hypercortisolism can directly affect the ovarian function of women and also inhibit the secretion of gonadotropin-releasing hormone by the hypothalamus, which in turn affects the secretion of follicle-stimulating hormone and luteinizing hormone by the pituitary gland, causing menstrual disorders and ovulation disorders in women, thus making pregnancy difficult, so pregnancy in combination with CS is rare (3). In 1953, Gemzell first reported CS of pregnancy (4). It is extremely rare for CS to occur at the same time as pregnancy (1). At present, about 250 cases have been reported worldwide (1, 5–7). During pregnancy, CS can lead to serious maternal and fetal complications, including spontaneous abortion, perinatal death, preterm delivery, hypertension, heart failure, diabetes, and opportunistic infection (1). In CS during pregnancy, the maternal mortality rate is as high as 5%, and the fetal mortality rate is as high as 25% (8). Due to the interference of maternal physiological changes during pregnancy, the clinical diagnosis of CS during pregnancy is prone to misdiagnosis and missed diagnosis (8, 9). Pregnancy complicated with CS is extremely disadvantageous to both mother and fetus (8), so early diagnosis and treatment are very important.

Antiphospholipid syndrome (APS) is an acquired thrombotic disease mediated by a variety of antiphospholipid components and phospholipid-binding protein antibodies, characterized by recurrent arteriovenous thrombosis and/or morbid pregnancy (early pregnancy abortion and middle and late fetal death) (10). As an autoimmune disease, the main symptoms of APS are recurrent thrombosis, abortion in early pregnancy, and stillbirth in the second and third trimesters, accompanied by persistently high titer-positive antiphospholipid antibodies (10, 11). The above symptoms can exist alone or together. Antiphospholipid antibodies include anticardiolipin antibodies, anti-β2 glycoprotein I antibodies, and lupus anticoagulants (11). According to the different clinical manifestations, the main clinical manifestation of thrombosis is called thrombotic APS (12), while the main clinical manifestation of adverse pregnancy outcomes is called obstetric APS (OAPS) (13). The diagnosis of OAPS should be based on the Sydney criteria (14). The concept of non-criteria OAPS (NC-OAPS) was proposed by Arachchillage et al. (15) in 2015. For pregnant women with only clinical or laboratory features, NC-OAPS should be diagnosed (16). Studies have shown that about 5% to 20% of reproductive women have clinical manifestations of APS, and if antiphospholipid antibodies positive patients do not take corresponding intervention or treatment, the pregnancy loss rate is as high as 24% to 60%, and perinatal pregnant women are prone to thrombus events, seriously endangering maternal lives (3). Therefore, early diagnosis, early prevention, and early treatment are of great clinical importance to improve the pregnancy outcome of patients with OAPS (3). It is important to emphasize that up to 10% of the general population may also have transient, low-titer antiphospholipid antibodies positivity (17), so overdiagnosis of OAPS should be avoided in clinical work. To our knowledge no cases of CS with OAPS and severe pre-eclampsia have been reported. We report the case of CS during pregnancy caused by an adrenal adenoma, complicated with OAPS, and discuss the clinical features, diagnosis, and treatment of the disease, as well as the maternal and fetal prognosis.

## 2 Clinical case

### 2.1 Patient’s past medical history

A 26-year-old woman, 20 weeks pregnant, went to the local hospital because of “systemic edema for 4 weeks”. In the 16th week of pregnancy, the patient presented with bilateral lower limb edema, measured blood pressure up to 175/110 mmHg (normal values (NV) are 90-139/60-89 mmHg, 1 mmHg = 0.133 kPa), reduced potassium to 2.48 mmol/L (NV: 3.5-5.5 mmol/L), cortisol (08:00) elevated to 27.15 μg/dl (NV: 4.82-19.50 μg/dl), cortisol (16:00) elevated to 27.11 μg/dl (NV: 2.47-11.90 μg/dl), cortisol (00:00) elevated to 30.23 μg/dl (NV: 0-6.7 μg/dl), and adrenocorticotropic hormone (ACTH) (08:00) decreased to 0.22 ng/dL (NV: 7.20-63.30 ng/dL). The patient had 4 spontaneous abortions in the past 4 years (the time of pregnancy loss was 11 weeks, 6 weeks, 7 weeks, and 4 weeks respectively). She denied a history of glucocorticoid use as well as family history of similar diseases, diabetes, and genetic diseases. The local hospital doctors gave the patient oral labetalol for hypertension, oral potassium chloride for hypokalemia, and oral aspirin for severe preeclampsia. The patient was treated with oral medication for up to one month, but the treatment was ineffective and she developed intractable hypertension and hypokalemia. Later, she was transferred to our hospital for further treatment.

### 2.2 Results of patient examination and preliminary diagnosis

**Figure 1:**
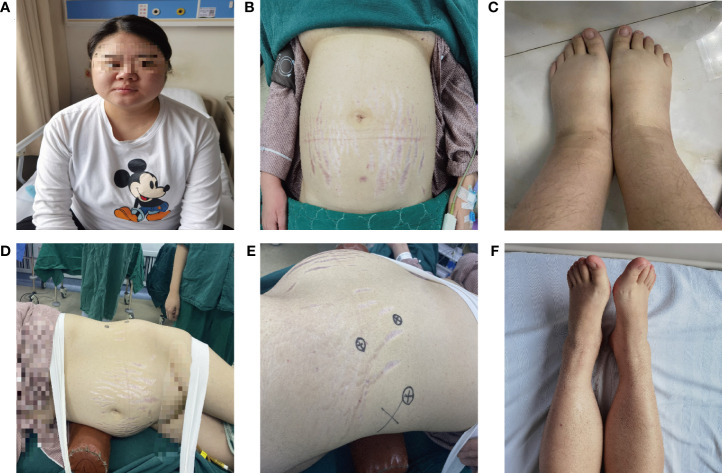
Clinical manifestations of CS with OAPS and severe PE during pregnancy. **(A–C)** The patient has clinical manifestations such as full moon face, centripetal obesity, purple striae on the abdomen, severe edema of the lower extremities; **(D–E)** Surgical posture of patients undergoing laparoscopic adrenalectomy; **(F)** The edema of the lower extremities was significantly relieved after surgical treatment.

**Figure 2:**
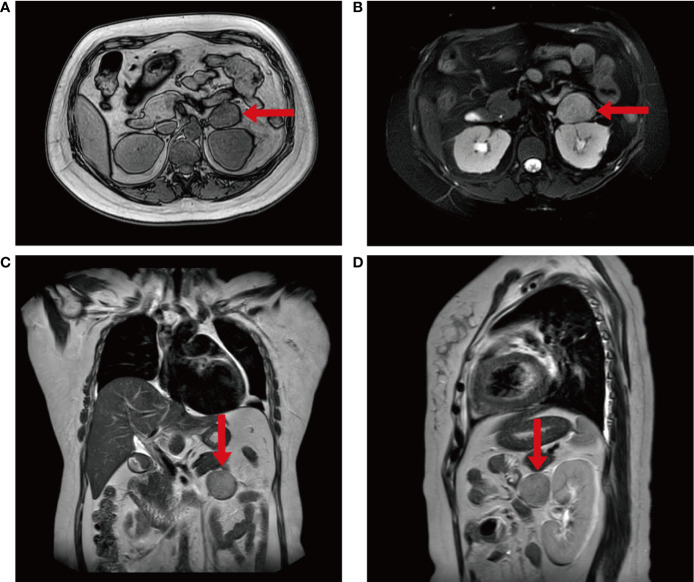
The imaging findings of the patient. **(A–D)** Plain MRI scan of the patient’s abdomen showed an abnormal signal mass in front of the left kidney, which was about 4.7x3.3x3.7cm in size and had a clear boundary.

The patient was referred to our hospital at 20 weeks of pregnancy. Physical examination showed: a body mass index of 29.3 kg/m^2^, full moon face, buffalo hump, acne visible on the chest and back, bulging abdomen, purple striae visible on the abdomen and inner thighs, and severe edema in both lower extremities (**Figure 1**). Hipercortisolism was diagnosed with two elevated 24-hour urinary cortisol levels (1190.5 ug/24 h and 1150.7 ug/24 h, normal value (NV): 12.3-103.5 ug/24 h) and two high late night salivary cortisol (37.80 nmol/L and 36.59 nmol/L, NV: <11.07 nmol/L). The patient’s ACTH (08:00) was suppressed (< 1.00 pg/ml, NV: 7.20-63.30 pg/ml) (**Table 1**). With the above results the patient was diagnosed with ACTH-independent CS. Blood pressure was measured up to 170/105 mmHg (NV: 90-139/60-89 mmHg) and blood potassium was reduced to 2.5 mmol/L (NV: 3.5-5.5 mmol/L). The patient had typical hypertension and hypokalemia, and to exclude primary aldosteronism, an aldosterone/renin activity ratio (ARR) test was performed, which showed normal results. The patient was positive for anti-cardiolipin antibodies: ACLA-IgG and ACLA-IgM. Coagulation function examination showed that fibrinogen increased to 4.41g/L (NV: 2-4g/L) and D-dimer increased to 1.04 μg/ml (NV: 0-1 μg/ml). Prothrombin time, activated partial thromboplastin time and thrombin time were normal. No abnormality was found in the antinuclear antibody spectrum, and diffuse connective tissue disease was excluded. Imaging examination showed that the intrauterine pregnancy, single live fetus, cephalic position, and fetal size were consistent with gestational weeks. Chromosome examination and prethrombotic gene test showed no abnormality. Adrenal color ultrasound revealed a mass in front of the left renal hilum, the nature of which was difficult to determine. The plain MRI scan of the abdomen showed a slightly longer T1W and slightly longer T2W abnormal signal mass in front of the left kidney, the size of which was about 47mm x 37mm (**Figure 2**). The patient was initially diagnosed with pregnancy combined with an adrenal tumor resulting in ACTH-independent CS, accompanied by NC-OAPS and severe pre-eclampsia.

**Table 1:** The changes of clinical indexes of this patient.

	Pre-surgery				Post-surgery					Normal range
	Admission to hospital	7days	3days	1day	Immediately after operation	1day	3days	7days	1 month	
Blood pressure	170/105*	142/90*	135/80*	130/82*	135/85	140/85	130/85	125/75	130/80	90-139/60-89mmHg
K(Potassium)	2.5*	3.2*	3.1*	3.3*	3.6	3.8	3.5	4.2	4.1	3.5-5.5mmol/L
Fetal heart rate	150	145	153	148	140	150	148	145	150	110-160 times per minute
Serum Cortisol (08:00)	34.88	34.61	34.99	34.61		60.22*	1.61*	0.97*	16.78	4.82-19.50μg/dl
Serum Cortisol (16:00)	34.75			35.21		14.27*	22.45*	10.52*	6.88	2.47-11.90μg/dl
Serum Cortisol (00:00)	34.99			34.33		63.44*	3.16*	3.16*	3.52	0-6.7μg/dl
LNSF	37.80	36.59								<11.07 nmol/L
24h UFC	1190.5	1150.7								12.3-103.5 ug/24 h
ACTH(08:00)	<1.00	<1.00				<1.00*		6.51*	20.32	7.20-63.30 ng/dL
Blood RBC	3.8	3.3		3.3	3.3	3.4		3.5	4.1	3.8-5.1×10^12/L
Hemoglobin	137	117		123	120	119		121	125	115-150g/L
D-dimer (DD)	1.04*		1.01	1.02	1.63	1.31*		1.00*	0.98*	0-1μg/ml

LNSF, late night salivary cortisol; 24h UFC, 24 h urine free cortisol; ACTH, adrenocorticotropic hormone; *After symptomatic treatment with related drugs.

### 2.3 Treatment

**Figure 3:**
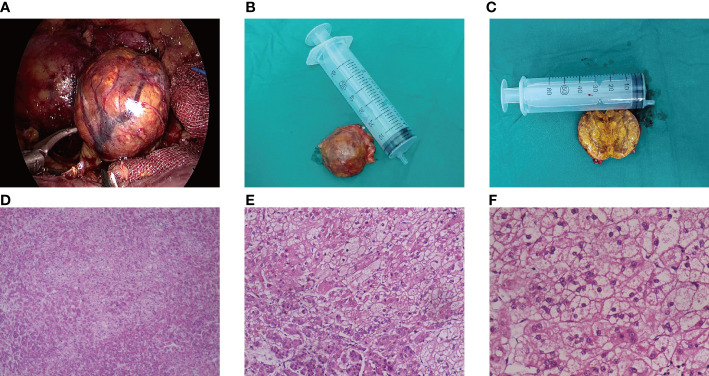
Pathological features of ACTH-independent CS during pregnancy caused by an adrenal adenoma. **(A–C)** The gross specimen of the tumor was a reddish-brown round mass, and the section of the tumor was golden after incision; **(D–F)** The tumor cells proliferated diffusely, and the cells of zona globularis, zona fasciculata, and zona reticularis could be seen.

The patient refused to take metyrapone to treat CS. The patient was treated with labetalol combined with nifedipine for hypertension, oral administration combined with intravenous potassium supplementation for hypokalemia, and low-dose aspirin combined with low molecular weight heparin for NC-OAPS and severe pre-eclampsia. Hypertension and hypokalemia improved with treatment, although hypokalemia did not normalize until after surgery. The patient had 4 previous spontaneous abortions and had a strong desire to continue the pregnancy. After preparation, retroperitoneal laparoscopic adrenal tumor resection was performed under general anesthesia at 23 weeks of pregnancy. During the operation, it was seen that the tumor originated from the left adrenal branch. The diameter of the tumor was about 4 cm. The capsule of the tumor was intact, and the surface was reddish brown (**Figure 3**). The operation time was about 50 minutes, and the blood loss was about 10 ml. The postoperative pathological result reported adrenocortical adenoma. The level of serum cortisol decreased rapidly after tumor resection. After the operation, the patients were treated with hormone therapy (prednisone, 15mg in the morning, 10mg in the afternoon, and hormone replacement therapy for 1 month). The patient lacked typical laboratory tests (the patient did not have two antiphospholipid antibodies with an interval between the results of the two tests of more than 12 weeks), so she was diagnosed with NC-OAPS and continued anticoagulant therapy with low-dose aspirin and low molecular weight heparin.

### 2.4 Follow-up

One month after the operation, oral dose of prednisone was stopped (after 24 hours of hormone withdrawal, the serum cortisol (08:00) was 16.78 μ g/dl, indicating the recovery of hypothalamus pituitary-adrenal axis). Hypertension and hypokalemia return to normal (**Table 1**). Anticardiolipin antibodies were reexamined at 35 weeks of gestation. The results showed that ACLA-IgG and ACLA-IgM were still positive and anticoagulant therapy continued. Combined with the previous history of four spontaneous abortions and the positive twice results of anticardiolipin antibodies with an interval of more than 12 weeks, the patient was finally diagnosed with OAPS. The patient underwent a cesarean section at 39 weeks of gestation to give birth to a healthy baby girl with an Apgar score of 10. The patient was successfully discharged from the hospital on the 3th day after delivery.

## 3 Discussion

### 3.1 Pregnancy complicated with CS

#### 3.1.1 Overview and status

Cushing’s syndrome is due to chronic exposure to high circulating cortisol levels (1). Cushing’s syndrome in pregnancy is a rare disease with an incidence of about 2/1000000 (18). At the same time, due to the interference of maternal physiological changes during pregnancy, doctors are prone to misdiagnose CS during pregnancy (18). Considering the safety factors of the fetus and mother, the choice of treatment for the disease is also faced with many contradictions.

#### 3.1.2 Pathogeny

According to the etiology, CS can be divided into ACTH-dependent and ACTH-independent CS (5). ACTH-dependent CS includes Cushing’s disease, ectopic ACTH syndrome (small cell lung cancer, thymic carcinoid, islet tumor), and ACTH-independent CS includes adrenocortical adenoma, adrenal nodular hyperplasia, and adrenal adenocarcinoma (19). There is a significant difference in the etiology of CS between pregnant women and non-pregnant women. In non-pregnant patients with CS, adrenal adenomas account for about 15% of cases, while in patients with pregnancy complicated with CS adrenal adenomas account for about 40% of them (5). In 2017, Caimari et al. (1) conducted a retrospective analysis of 263 cases of pregnancy CS. The results showed that adrenal adenoma was the main cause of CS during pregnancy, and patients were more likely to develop gestational diabetes mellitus, gestational hypertension, and preeclampsia (1). In addition, the diagnosis of CS during pregnancy is also associated with poor fetal prognosis (1). During normal pregnancy, a series of physiological changes have taken place in the hypothalamus-pituitary-adrenal axis, such as the production of corticotropin and corticotropin-releasing hormone in the placenta, the increase of maternal ACTH and free cortisol, and the change of negative feedback mechanism regulating the secretion of ATCH in hypothalamus-pituitary-adrenal axis, which leads to the increase of cortisol concentration in serum and urine of pregnant women, but can still maintain circadian rhythm (20, 21). In some cases, such as pregnancy-induced CS and other cases of CS during pregnancy ectopic or aberrant LH/hCG-receptors expressed in the adrenal gland have been suggested to be involved in the pathogenesis (20, 21). The case reported in this article is an ACTH-independent CS caused by an adrenocortical adenoma, which is the most common type of CS during pregnancy.

#### 3.1.3 Clinical manifestation

Pregnancy complicated with CS can be characterized by centripetal obesity, edema, abdominal purple striae, hypertension, and hyperglycemia, and normal pregnant women can show similar symptoms during pregnancy, except abdominal purple striae. In a healthy pregnancy without CS non-violaceous striae are common encountered and lack discriminatory value (1). Discriminatory signs that rise suspicion of CS includes purple striae (particularly other sites than the abdomen), thin skin, easy bruising, proximal myopathy, and hypokalemia (1, 22). Other features that are not typical see in pregnancy are pathological fractures, osteoporosis, and nephrolithiasis (1, 23, 24). Study analysis showed that compared with normal pregnancy, the incidence of complications in patients with CS during pregnancy and the fetus was significantly higher, including preterm delivery, stillbirth, spontaneous abortion, fetal intrauterine growth restriction, adrenal dysfunction, fetal malformation, and ventricular hemorrhage (1, 7, 25). Hypokalemia occurred frequently in this patient before the operation. Primary hyperaldosteronism was excluded by the aldosterone/renin activity ratio. Severe hypokalemia in pregnancy is more frequent in ectopic CS, adrenal carcinoma, and pregnancy-induced CS (1).

#### 3.1.4 Diagnosis

In addition to the typical clinical manifestations, the diagnosis of CS first needs to determine whether there is high cortisol and whether the circadian rhythm of cortisol disappears (26, 27). Pregnancy itself can change the regulation of the hypothalamus-pituitary-adrenal axis (28). In the middle and third trimester of pregnancy, the synthesis and release of ACTH and corticotropin-releasing hormone in the placenta increase, so serum cortisol and urinary free cortisol can increase by 2-3 times of normal value (26). When urinary free cortisol increases to more than 3 times the upper normal range, the possibility of CS should be considered (7, 23). For non-pregnant women, it is feasible to make a definite diagnosis by dexamethasone inhibition test and corticotropin-releasing hormone stimulation test (29, 30). However, due to the physiological changes of the hypothalamus-pituitary-adrenal axis during pregnancy, the dexamethasone inhibition test can not inhibit cortisol secretion during normal pregnancy, which can easily cause false positive results (30, 31). The dexamethasone suppression test is not reliable during pregnancy due to increased levels of cortisol and cortisol-binding globulin (30). Uterine contractions caused by corticotropin-releasing hormones may increase the risk of preterm delivery (30). In normal pregnancy, the level of cortisol increases, but cortisol has a circadian rhythm, so CS can be diagnosed by the disappearance of the normal rhythm of cortisol (30). The more accurate and convenient method is to measure salivary cortisol (30, 31). The second step is to identify the cause (23). The determination of blood ACTH level can simply distinguish between ACTH-dependent CS and ACTH-independent CS, and the former has an increase in ACTH (23, 24). For pregnant patients, the use of radiation imaging examination is limited (1). Adrenal MRI without contrast is the imaging of choice, although adrenal ultrasound can be helpful in some cases (1).

#### 3.1.5 Treatment

Treatment of CS in pregnancy includes pharmacological and surgical treatment (1). Caimari et al. (1) summarized the treatment outcomes of different treatment options for patients with CS in pregnancy and showed that the surgical treatment group was superior to the pharmacological treatment group than to the control group without any treatment. In 2022, Hamblin et al. (23) summarized the drugs that have been used in patients with CS, including metyrapone, ketoconazole, cabergoline, mitotane, and cyproheptadine. However, in view of pregnancy complicated with CS, the use of the above drugs in patients with pregnancy complicated with CS will be limited and should be treated individually according to the specific condition of the patient (1, 32–37). Excess cortisol secretion suppresses corticotropin releasing hormone and ACTH and causes atrophy of the contralateral adrenal gland, so adrenal insufficiency or post-operative hypocortisolism is a predictor of cure (1, 24). The best treatment strategy for pregnancy CS caused by an adrenal tumor is surgical resection of the adrenal tumor (1, 19). Surgical in the second trimester is recommended to reduce the risk of maternal and fetal complications (1, 19).

### 3.2 Obstetrical antiphospholipid syndrome

#### 3.2.1 Summary

Antiphospholipid syndrome is a common systemic autoimmune disease, which is related to obstetrical complications and thrombus events associated with antiphospholipid antibodies (10, 38). Antiphospholipid syndrome is common in women of childbearing age, with the highest incidence during pregnancy (10, 38). According to the different clinical manifestations, the main clinical manifestation of thrombosis is called thrombotic APS (12), while the main clinical manifestation of adverse pregnancy outcomes is called OAPS (13). Adverse pregnancy outcomes caused by APS include recurrent abortion, preterm delivery, fetal growth restriction, and pre-eclampsia (2, 10, 13, 39). Pre-eclampsia is a common complication of pregnancy, with hypertension and proteinuria after 20 weeks of pregnancy as the main clinical manifestations (10, 39). The condition of APS complicated with PE is more serious, even after standard anticoagulant therapy, it is still easy to have adverse pregnancy outcome (39). At present, the diagnosis and treatment of OAPS have been widely concerned by clinicians, but there is still difficulties in the treatment of NC-OAPS, refractory OAPS, and OAPS with thrombocytopenia (11, 13, 15).

#### 3.2.2 Pathogenesis and clinical manifestation

The body of pregnant women with APS is in a prethrombotic state (40). The function of coagulation, anticoagulation, and the fibrinolytic system is dysfunctional, and the body is in a persistent hypercoagulable state, which selectively affects uterine-placental circulation and leads to placental microcirculation disturbance, causing ischemic damage to placental tissue, leading to chorionic infarction and decidual fibrin-like necrosis, and hindering material exchange between fetus and mother (13, 40). To promote the occurrence of a series of pathological pregnancies such as abortion, premature delivery, fetal growth restriction, and pre-eclampsia (13, 40). The pathogenesis of obstetrical APS is complex and diverse, which is not completely clear at present (40). We summarized the possible pathogenesis (40–43) (1): Antiphospholipid antibody interacts with β 2 glycoprotein I to induce blood coagulation (2); Antiphospholipid antibodies recognize phospholipid binding proteins expressed on platelets and activate platelet coagulation (3); Antiphospholipid antibodies induce the release of cytokines through leukocytes and monocytes and promote blood coagulation (4); Antiphospholipid antibodies recognize anti-β 2 glycoprotein I antibodies to trigger high levels of proinflammatory cytokines produced by extravillous trophoblasts in early pregnancy and inhibit the spontaneous migration of trophoblasts (5); Antiphospholipid antibodies activate the complement system, resulting in the release of anti-angiogenic factors and affecting the angiogenic factors needed for normal pregnancy (6); Antiphospholipid antibodies inhibit the growth of syncytial trophoblast, resulting in an increase in syncytial trophoblast death and a decrease in human chorionic gonadotropin production; and (7) Antiphospholipid antibodies cause placental vascular remodeling disorder, placental superficial implantation and tissue hypoxia lead to placental diseases.

#### 3.2.3 Diagnosis and differential diagnosis

In 1999, Wilson et al. (44) proposed the classification criteria for APS. In 2006, Miyakis et al. (14) revised the original definition and developed the Sydney classification standard, which is still used today. Obstetrical antiphospholipid antibody syndrome is a kind of APS, which is related to various obstetric complications (14, 45, 46). The diagnostic criteria are shown in **Table 2**. Arachchillage et al. (15) put forward the concept of NC-OAPS in 2015, which should be diagnosed as NC-OAPS for pregnant women with only clinical manifestations or laboratory features. However, the Sydney standard has some limitations in the diagnosis of APS (16). Due to the limitations of the types and methods of antibody testing, it is easy to miss for some patients who have clinical manifestations of APS but can not meet their laboratory criteria (16). At present, anti-phospholipid antibodies include anticardiolipin antibodies, anti-β2 glycoprotein I antibodies, and lupus anticoagulants (16, 46). Only the detection of these three kinds of antibodies has some limitations (16). However, some women have a history of recurrent adverse obstetric outcomes (16). Cases with incomplete clinical or laboratory data are classified as obstetric morbidity APS (OMAPS) and NC-OAPS (16, 46) (**Table 3**). Recent studies on the pathogenesis of APS and clinical trials have shown that antiphospholipid antibodies exist outside some classification criteria and are associated with thrombotic events and morbid pregnancy events, including (1) Anti-prothrombin antibodies (47) (2); Anti-β2GPI domain1antibodies (48) (3); IgA anti-β 2GPI antibody and IgA anticardiolipin antibody (49) (4); Anti-cardiolipin/vimentin antibodies (50) (5); Anti-annexin A2 antibodies/anti-annexin A5 antibodies (51) (6); Antiphospholipid antigen antibody (52) (7); Anti-protein C/protein S antibody (53). In addition, some patients with APS may also have clinical manifestations outside the classification criteria, such as superficial venous thrombosis, thrombocytopenia, renal microvascular disease, cardiac valvular disease, migraine, chorea, epilepsy, and myelitis (50, 54). In recent years, studies have found that some antibodies and clinical manifestations outside the classification criteria are closely related to APS, which is expected to provide a basis for new treatments.

**Table 2:** Comparison of OAPS and NC-OAPS diagnostic criteria.

	Clinical Criteria	Laboratory criteria
OAPS	1. ≥3 consecutive miscarriages before week 10 of gestation 2. At least one fetal loss after week 10 of gestation 3. At least one premature birth before week 34 of gestation due to PE/eclampsia or placental insufficiency	1. Two LA positive tests at least 12 weeks apart 2. Two IgG or IgM aCL positive tests at least 12 weeks apart 3. Two IgG or IgM aβ2GPI positive tests at least 12 weeks apart
NC-OAPS		1. Positivity for LA, aCL or aβ2GPI only detected once 2. Low positive IgG/IgM aCL or aβ2GPI titers 3. Persistent positivity for non-criteria aPL, including IgA-aCL and aβ2GPI 4. Resistance to Annexin A5 anticoagulant activity 5. Thrombocytopenia

aCL, IgG/IgM anticardiolipin antibodies; aβ2GPI, IgG/IgM antiβ2-glycoprotein I antibodies; aPL, antiphospholipid antibodies; LA, lupus anticoagulant; OAPS, obstetric antiphospholipid syndrome; NC-OAPS, non-criteria OAPS.

**Table 3:** Comparison of OAPS and MOAPS diagnostic criteria.

	Laboratory criteria	Clinical criteria
OAPS	1. Two LA positive tests at least 12 weeks apart 2. Two IgG or IgM aCL positive tests at least 12 weeks apart 3. Two IgG or IgM aβ2GPI positive tests at least 12 weeks apart	1. ≥3 consecutive miscarriages before week 10 of gestation 2. At least one fetal loss after week 10 of gestation 3. At least one premature birth before week 34 of gestation due to PE/eclampsia or placental insufficiency
OMAPS		1. Two consecutive unexplained miscarriages of well-formed embryos 2. Three or more non-consecutive miscarriages of well-formed embryos 3. PE/eclampsia after week 34 of gestation or at puerperium 4. Placental abruption 5. Late premature birth 6. Premature rupture of membranes 7. Unexplained recurrent implantation failure in in vitro fertilization (failure of at least 3 embryo transfer)

aCL, IgG/IgM anticardiolipin antibodies; aβ2GPI, IgG/IgM antiβ2-glycoprotein I antibodies; aPL, antiphospholipid antibodies; LA, lupus anticoagulant; OAPS, obstetric antiphospholipid syndrome; OMAPS, obstetric morbidity antiphospholipid syndrome; PE, pre-eclampsia.

#### 3.2.4 Treatment

The treatment of OAPS is to minimize the risk of maternal and fetal complications, including thrombosis, abortion, stillbirth, pre-eclampsia, placental dysfunction, and iatrogenic preterm delivery (55, 56). The main clinical treatments are antiplatelet therapy, anticoagulant therapy, and immunomodulator therapy (57). Without proper treatment, patients may have systemic inflammation, multiple organ failure, and even die (57). The main drugs currently used in clinical practice include heparin, aspirin, corticosteroids, immunoglobulin, and hydroxychloroquine (57–59).

Treatment before pregnancy: Patients with planned pregnancy are advised to receive prophylactic treatment with low-dose aspirin (75~100mg/d) (60). Hydroxychloroquine (200~400mg/d) should be comprehensively considered according to the antibody titer of OAPS patients with failure of routine aspirin treatment, previous history of thrombosis, or OAPS complicated with other systemic immune diseases (57, 59, 60). NC-OAPS and OAPS have the same before pregnancy treatment regimen (59, 60).

Treatment during pregnancy: For patients with OAPS, it is recommended to combine low-dose aspirin (50~100mg/d) with low molecular weight heparin (5000U/d) (15, 16). For NC-OAPS, it is recommended to use low-dose aspirin (50~100mg/d) or in combination with low molecular weight heparin (5000U/d) according to the patient’s own conditions, such as antiphospholipid antibody spectrum, previous history of thrombosis and pregnancy loss (15, 16). Some studies have shown that both OAPS and NC-OAPS have the same potential risk of adverse pregnancy outcomes, which can lead to maternal-fetal complications mediated by antiphospholipid antibodies (15, 61). Pregnancy outcomes of NC-OAPS can be significantly improved with treatment (15, 61).

Postpartum treatment: The hypercoagulable state can last up to 12 weeks after delivery, and in the postpartum patients with OAPS have mainly venous thrombosis events (16, 38). For patients with positive antiphospholipid antibodies, anticoagulant therapy is recommended until 6-12 weeks postpartum to reduce the risk of postpartum thrombotic events (59–61).

As far as we know, no case of CS with OAPS has been reported. Cushing’s syndrome is associated with hypercoagulable state, leading to an increased risk of venous thromboembolism (VTE) (62). Among untreated CS patients, the incidence of VTE was 18 times higher than that of the general population, and the risk of VTE decreased significantly after surgical resection of adrenal tumors (63). The hypercoagulable state of CS is not fully understood, and some studies suggest that it is caused by the imbalance between activity of procoagulant and anticoagulant pathways (62). Functional analysis showed that the partially activated thromboplastin time was shortened and the thrombolysis time was increased in patients with CS (63, 64). This may aggravate the coagulation dysfunction in patients with OAPS, affect uterine and placental circulation, and lead to a series of adverse pregnancy outcomes (11, 16, 63, 64). There is no standard scheme for preoperative or postoperative thrombus prevention in patients with CS complicated with OAPS.

## 4 Conclusion

To our knowledge, we report for the first time a case of pregnancy combined with adrenal adenoma causing ACTH-independent CS, accompanied by OAPS and severe pre-eclampsia. Cushing’s syndrome caused by adrenal adenoma in pregnancy is rare and difficult to diagnose. Surgery is the first choice for the treatment of CS during pregnancy, and the recommended time of operation is the second trimester of pregnancy. Obstetric antiphospholipid syndrome is a subtype of APS. If OAPS patients do not take corresponding treatment, their perinatal pregnant women are prone to thrombus events and adverse pregnancy outcomes, seriously endangering the lives of pregnant women. The effect of hypercoagulable state of CS on OPAS is unknown. In view of the rarity of the disease and the difficulty of treatment of concomitant diseases, it is necessary to establish a multidisciplinary team, including endocrinology, obstetrics and gynecology, urology, and rheumatism immunology department, to develop an individualized treatment for patients. Strive for early detection, and timely intervention of the disease, and then maximize the maternal and fetal prognosis.

## Data availability statement

The original contributions presented in the study are included in the article/supplementary material. Further inquiries can be directed to the corresponding author.

## Ethics statement

The studies involving human participants were reviewed and approved by the Ethics Committee of the Affiliated Hospital of Guizhou Medical University. The patients/participants provided their written informed consent to participate in this study. Written informed consent was obtained from the individuals for the publication of any identifiable images or data included in this article.

## Author contributions

KT and SX: study concept and design. SX, ML, JX, BC, WZ, WL, TH, YY, CZ, ZP, and KH: clinical data collection. SX: draft. KT, ML, and JX: critical revision of manuscripts. All authors contributed to the article and approved the submitted version.
